# Prevalence, risk factors, and clinical manifestations of schistosomiasis among school children in the White Nile River basin, Sudan

**DOI:** 10.1186/s13071-014-0478-6

**Published:** 2014-10-15

**Authors:** Hassan Ahmed Hassan Ahmed Ismail, Sung-Tae Hong, Azza Tag Eldin Bashir Babiker, Randa Mohamed Abd Elgadir Hassan, Mohammed Ahmed Zakaria Sulaiman, Hoo-Gn Jeong, Woo-Hyun Kong, Soon-Hyung Lee, Han-Ik Cho, Hae-Sung Nam, Chung Hyeon Oh, Young-Ha Lee

**Affiliations:** Departments of Infection Biology, Chungnam National University School of Medicine, Daejeon, 301-131 Korea; Department of Parasitology and Tropical Medicine, Seoul National University College of Medicine, Seoul, 110-799 Korea; Schistosomiasis, Lymphatic Filariasis & Sleeping Sickness Control Program, Federal Ministry of Health, Khartoum, Sudan; Schistosomiasis Research Laboratory, Department of Zoology, Faculty of Science, University of Khartoum, Khartoum, Sudan; Korea Association of Health Promotion (KAHP), Seoul, 157-705 Korea; Department of Preventive Medicine, Chungnam National University School of Medicine, Daejeon, 301-131 Korea; Korea International Cooperation Agency (KOICA), Soengnam, 461-833 Korea

**Keywords:** *Schistosoma haematobium*, *Schistosoma mansoni*, Egg positive rate, Infection intensity, Water-contact pattern, White Nile River basin of Sudan

## Abstract

**Background:**

We investigated the prevalence, risk factors, and clinical manifestations of schistosomiasis in White Nile State, Sudan, to determine the local characteristics of schistosomiasis in the White Nile River basin.

**Methods:**

Urine and stool samples were collected from 338 students (176 boys, 162 girls) at three primary schools and were examined using the urine filtration method and the Kato-Katz technique, respectively. Of the students, 200 were interviewed using a semi-structured questionnaire to assess water-contact patterns and health conditions related with urinary schistosomiasis.

**Results:**

Of the 338 students, egg-positive rates for *S. haematobium* and *S. mansoni* were 45.0% and 5.9%, respectively, and 4.4% were mixed. The intensities of *S. haematobium* and *S. mansoni* infection were 1.091 ± 0.744 log EP10 (eggs per 10 mL of urine, mean ± SD = 57 ± 172 EP10) and 1.787 ± 0.844 log EPG (eggs per gram of stool, mean ± SD = 156 ± 176 EPG), respectively. The prevalence and intensity of *S. haematobium* infection differed significantly among the three schools, but not by gender or age. Urinary schistosomiasis was significantly associated with the frequencies of contaminated water contact, taking baths, swimming, and wading the stream; however, frequencies of these events were not significantly correlated with infection intensity. Self-reported hematuria and dysuria also correlated significantly with urinary schistosomiasis.

**Conclusions:**

The overall prevalence of schistosomiasis, especially urinary schistosomiasis, is high in the White Nile River basin, Sudan, and is closely associated with frequencies of water contact, taking baths, swimming, and wading the stream. We strongly recommend implementation of an integrated schistosomiasis control program in this area.

## Background

Schistosomiasis, one of the most prevalent neglected tropical diseases (NTDs), remains as a public health problem in many developing countries in the tropics and subtropics, and ~700 million people worldwide are at risk of this infection [1,2]. Over 90% of the disease is currently found in sub-Saharan Africa, where more than 200,000 deaths are attributed to schistosomiasis annually [3,4]. There are several examples of dramatic increases in the prevalence of schistosomiasis as a result of irrigation project construction in sub-Saharan Africa [5]. The World Health Organization (WHO) estimated that the number of countries considered as endemic for schistosomiasis was 78 in 2011, and ~243 million people require preventative chemotherapy, including 111 million school-age children, of which 226 million are in Africa [3]. However, only ~28 million people received treatment, which is only 10.2% coverage of the global requirement for schistosomiasis treatment [3].

Schistosomiasis is a parasitic disease caused by blood vessel-dwelling flukes of the genus *Schistosoma*. There are several species in the genus but primarily *S. haematobium* (causes urinary schistosomiasis), *S. mansoni*, and *S. japonicum* (both cause intestinal schistosomiasis) infect humans. Humans are usually infected by cercarial invasion through the skin when they come into contact with contaminated freshwater during daily life [1]. In the endemic areas, children, women, fishermen, and farmers in irrigation channels are often infected with schistosomes. Urinary schistosomiasis is characterized by hematuria as a classical sign, and is associated with bladder and urethral fibrosis and hydronephrosis that are commonly seen in chronic cases, while bladder cancer is a possible late-stage complication [6]. Clinical manifestations of intestinal schistosomiasis include abdominal pain, diarrhea, and blood in the stool. In advanced cases, hepatosplenomegaly is common and is repeatedly associated with ascites and other signs of portal hypertension [1].

Sudan has wide river basin areas, due to the crossings of the Blue Nile, White Nile, and Nile Rivers, and had a large irrigated agriculture sector along the banks of these rivers. Due to this geographical environment, schistosomiasis has affected many people of Sudan for many centuries, especially in the major irrigation systems in the Gezira area between the Blue and White Nile Rivers [7,8]. To date, schistosomiasis is the most prevalent parasitic disease in Sudan, and there have been some epidemiological studies on human schistosomiasis in the Gezira Managil area, Southern Kordofan, and South Darfur, Sudan [7-14]. The White Nile River is across the White Nile State. The slow current of the White Nile River and the presence of the dense grasses and vegetation in the river create a good environment for intermediate host breeding and growth. Furthermore, there are few sanitary and clean water-supply facilities in White Nile State. It was reported that White Nile State was one of the endemic regions of schistosomiasis [8]. However, it is difficult to find recent reports, published within 10 years, about the prevalence of schistosomiasis of residents who live in White Nile River basin of White Nile State. Furthermore, there has been no reports about the risk factors for transmission of schistosomiasis in Sudan. Therefore, in order to evaluate the prevalence, risk factors for transmission, and clinical manifestations of schistosomiasis in the White Nile River basin, Sudan, we conducted urine and stool examinations as well as questionnaire surveys at three primary schools along the White Nile River basin in Sudan.

## Methods

### Ethical statement

This study protocol was reviewed and approved by the institutional review board of the Korea Association of Health Promotion (Acceptance No. 10-C-05) and was also approved by the National Control Program for Schistosomiasis and Soil-Transmitted Helminthes, Federal Ministry of Health, Sudan. Before doing the survey at each school, informed verbal and/or formal written consent was obtained from each child in the presence of school teachers.

### The surveyed areas and population

**Figure 1:**
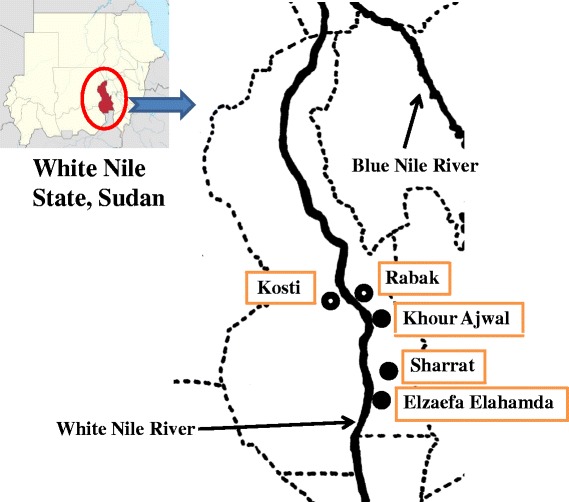
**Location of study areas in the White Nile River basin, White Nile State, Sudan.** The colored area indicates White Nile State, Sudan. The White Nile River runs inside the state and crosses the whole state.

The purpose of this survey was to evaluate the prevalence of schistosome infection among the primary school children in White Nile State. Thus, three primary schools were selected in the White Nile River basin in White Nile State, Sudan (Figure 1). The local activities of inhabitants were based on agriculture. In total 338 (176 boys, 162 girls) were enrolled from three primary schools at Khour Ajwal, Elzaefa Elahamda, and Sharrat villages (Table 1). All schools were located adjacent to the White Nile River, where the houses in the villages were built with mud bricks. Khou Ajwal and Elzaefa Elahamda schools are located within 1 km from the border of White Nile River, whereas Sharrat school is located about 3 km form the border of the River. They consisted of 47.0% 7–9-year-olds (159 students), 33.7% 10–12-year-olds (114 students), and 19.2% 13–15-year-olds (65 students). Their mean age was 10.0 (range, 7–15) years old; 52.1% (176) were boys and 47.9% (162) girls.

**Table 1:** **Lists of surveyed schools, school children and samples for the examination of schistosomiasis in White Nile State, Sudan**

**School name**	**Sex**	**No. of exam (%)**	**Age group (Years old)**				**No. of samples**		
			**7-9**	**10-12**	**13-15**	**Total**	**Urine**	**Stool**	**Questionnaire**
Khour Ajwal	Boys	47	22	14	11	47	47	47	47
	Girls	78	28	28	22	78	78	78	78
	**Subtotal**	**125 (37.0)**	**50**	**42**	**33**	**125**	**125**	**125**	**125**
Elzaefa Elahamda	Boys	55	**25**	22	8	55	55	55	40
	Girls	29	17	**7**	5	29	29	29	10
	**Subtotal**	**84 (24.9)**	**42**	**29**	**13**	**84**	**84**	**84**	**50**
Sharrat	Boys	74	35	25	14	74	74	74	14
	Girls	55	32	18	5	55	55	55	11
	**Subtotal**	**129 (38.2)**	**67**	**43**	**19**	**129**	**129**	**129**	**25**
Total	Boys	176 (52.1)	82	61	33	176	176	176	101
	Girls	162 (47.9)	77	53	32	162	162	162	99
	**Total**	**338 (100.0)**	**159 (47.0)**	**114 (33.7)**	**65 (19.2)**	**338 (100.0)**	**338 (100.0)**	**338 (100.0)**	**200 (59.2)**

### Parasitological examination

Parasitological surveys were undertaken from April, 2009 to February, 2010. The urine and stool samples were collected and immediately transferred to the parasitological laboratory in Rabak, White Nile State, Sudan. Only one sample each of urine and stool was taken per child. We first observed whether the urine samples showed occult or gross hematuria, and then the urine samples were screened for *S. haematobium* eggs by a filtration method [15]. The filtration device was composed of a plastic filter holder with a nylon filter (pore size, 12.0 μm; Millipore, Ireland), fixed by a rubber O-ring that prevented urine from bypassing the filter. Urine (10 mL) was filtered forcibly through the filter membrane with a syringe. Eggs of *S. haematobium* were filtered and counted per 10 mL of urine (EP10) under a light microscope. Infection intensities were classified into two categories: light (EP10 < 50) and heavy infection (EP10 ≥ 50) [16].

The collected stool samples were diagnosed parasitologically using the Kato-Katz technique [17]. The fecal materials were examined under a microscope, and counts were multiplied by 24 to provide total estimated egg counts. The infection intensity of *S. mansoni* was classified according to the number of eggs per gram of stool (EPG): light (EPG <100), moderate (100 ≤ EPG <400), or high (EPG ≥400) [18]. We also examined the other parasites beside schistosomes from the stool samples.

### Interviews of school children on water-contact patterns and health conditions

The semi-structured questionnaires consisted of two parts: one for water-contact patterns influencing transmission of schistosomiasis, and the other for information about health conditions related to schistosomiasis. Teachers or health officials explained the process to the students thoroughly, and then the school children were interviewed individually, and their verbal responses were written down. Interviews were carried out with 125 students at Khour Ajwal school, 50 students at Elzaefa Elahamda school, and 25 students at Sharrat school, depending on the availability of interviewers.

### Statistical analyses

Data were analyzed using the SPSS software (ver. 16.0; Chicago, IL, USA). Due to the deviation from normality distribution, infection intensity in terms of EP10 and EPG was transformed into logarithm, namely log EP10 or log EPG. The differences in continuous variables among groups were tested using a two-tailed Mann-Whitney *U* test, Kruskal-Wallis test, and Student’s *t* test. Categorical variables were tested using the *χ*^2^ test. Logistic regression analysis was used to assess the association between study variables and *S. haematobium* and *S. mansoni* infection. Odds ratios (OR) and 95% confidence intervals (CI) were calculated. Differences among groups were considered significant at *P* <0.05.

## Results

### Overall prevalence of schistosomiasis

**Figure 2:**
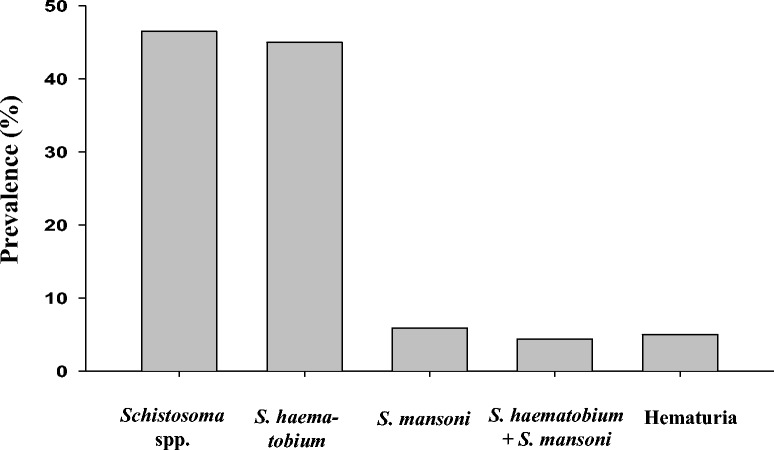
**Positive rates of** ***Schistosoma haematobium*** **and/or** ***S. mansoni*** **infections and hematuria.** Of the 338 students, 46.5% were infected by *S. haematobium* or *S. mansoni,* and 4.4% had mixed infections. The egg-positive rates for *S. haematobium* and *S. mansoni* were 45.0% and 5.9%, respectively. Hematuria was seen in 5.0% of the students.

As shown in Table 2 and Figure 2, 157 of 338 (46.5%) students were found to be infected by *S. haematobium* or *S. mansoni*, and 4.4% of them had mixed infections. Seventeen children (5.0%) from two schools (Khour Ajwal and Elzaefe Elahamda) had visible hematuria. The egg-positive rates for *S. haematobium* and *S. mansoni* were 45.0% (152 cases) and 5.9% (20 cases), respectively. The egg-positive rate of *Schistosoma* species in boys and girls were 48.9% (86 cases) and 43.8% (71 cases), respectively, and schistosome egg-positive rates were not significant differences of between sexes (0.213 < *P* <0.590). The egg-positive rates by age group were 47.8% in 7–9-year-olds (76/159), 44.7% in 10–12-year-olds (51/114), and 46.2% in 13–15-year-olds (30/65; Table 2). The egg-positive rates were not different between groups by age (*P >*0.05), but did differ significantly by village: 60.0% in Khour Ajwal, 73.8% in Elzaefa Elahamda, and 14.7% in Sharrat school (*P* <0.05). All students infected with schistosomes were treated with praziquantel at 40 mg/kg. From the stool samples We also found 6 cases of *Hymenolepis nana*, 1 case of *H. diminuta* and 3 cases of *Entamoeba coli*, beside schistosome.

**Table 2:** **Results of positive cases of** ***Schistosoma*** **species based on urine and stool examination according to school and sex in White Nile State, Sudan**

**School name**	**Sex**	**No. of exam. (%)**	***Schistosoma*** **egg positive cases by age group (%)**				**OR (95% ** **CI)**	***P-*** **value**
			**7-9**	**10-12**	**13-15**	**Total**		
Khour Ajwal	Boys	47	16	9	5	30	1.228 (0.582-2.590)	0.590
	Girls	78	16	17	13	46	1	
	**Subtotal**	**125**	**33**	**26**	**18**	**76 (60.8)**		
Elzaefa Elahamda	Boys	55	20	15	8	43	1.886 (0.695-5.116)	0.213
	Girls	29	11	5	3	19	1	
	**Subtotal**	**84**	**31**	**20**	**11**	**62 (73.8)**		
Sharrat	Boys	74	8	4	1	13	1.740 (0.616-4.913)	0.295
	Girls	55	5	1	0	6	1	
	**Subtotal**	**129**	**13**	**5**	**1**	**19 (14.7)**		
Total	Boys	176	44	28	14	86 (48.9)	1.225 (0.798-1.880)	0.354
	Girls	162	32	23	16	71 (43.8)	1	
	**Total**	**338 (100.0)**	**76 (47.8)**	**51 (44.7)**	**30 (46.2)**	**157 (46.5)**		

OR, Odds ratio.

CI, confidence interval.

### Analysis of egg-positive cases of *S. haematobium*

The egg-positive rate of *S. haematobium* was 45.0% (152 cases), and 15 of them were also infected with *S. mansoni* (double infection rate, 4.4%; Tables 2 and 3, Figure 2). The *S. haematobium* egg-positive rates of boys and girls were 48.9% (86/176) and 40.7% (66/162; *P* = 0.134), respectively. The egg-positive rates by age groups were 45.6% in 7–9-year-olds, 43.0% in 10–12-year-olds, and 44.6% in 13–15-year-olds (0.560 < *P <*0.820). According to school, the egg-positive rates for *S. haematobium* in Khour Ajwal, Elzaefa Elahamda, and Sharrat schools were 56.8%, 73.8%, and 14.7%, respectively (*P* <0.001).

**Table 3:** **Univariate analysis of factors associated with** ***S. haematobium*** **infection among school children who participated in this study (n = 338)**

**Variable**	**Category**	**No. exam.**	**No. positive (%)**	**OR (95% ** **CI)**	***P-*** **value by logistic regression**	**Intensity of positive cases**	
						**Mean ± S.D. (log EP10)**	***P-*** **value**
Age in years	7-9	159	74 (45.6)	1		1.181 ± 0.736	0.249
	10-12	114	49 (43.0)	0.87 (0.53-1.41)	0.560	1.061 ± 0.800	
	13-15	65	29 (44.6)	0.93 (0.52-1.65)	0.832	0.914 ± 0.654	
Sex	Boys	176	86 (48.9)	1		1.147 ± 0.732	0.296
	Girls	162	66 (40.7)	0.72 (0.47-1.11)	0.134	1.019 ± 0.760	
School	Khour Ajwal	125	71 (56.8)	7.61 (4.17-13.90)	*<0.001*	0.936 ± 0.824	*0.001*
	Elzaefa Elahamda	84	62 (73.8)	16.32 (8.20-32.47)	*<0.001*	1.355 ± 0.634	
	Sharrat	129	19 (14.7)	1		0.810 ± 0.483	

OR, Odds ratio.

CI, confidence interval.

The intensities of *S. haematobium-*infected school children were 1.091 ± 0.744 log EP10 (range, 1–1,755 EP10; mean ± SD = 57 ± 172 EP10; Table 3). Of these infected school children, 76.3% had light infection (EP10 < 50) and 23.7% were considered to be heavily infected. The mean intensity in boys was 1.147 ± 0.732 log EP10 (range, 1–1,755 EP10) while that of girls was 1.019 ± 0.760 log EP10 (range, 1–570 EP10; *P* = 0.296). According to age group, the highest intensity was seen in the 7–9 year age group (1.18 ± 0.736 log EP10; range, 1–864 EP10), followed by 10–12 years and 13–15 years (*P* = 0.249). The intensity difference between schools was significant, with the highest burden of 1.355 ± 0.634 log EP10 (range, 1-864 EP10) at Elzaefa Elahamda school (*P* = 0.001).

### Analysis of egg-positive cases of *S. mansoni*

The egg-positive rate for *S. mansoni* was 5.9% (20 cases), and 15 of them were also infected with *S. haematobium* (Tables 2, 4). The *S. mansoni* egg-positive rate in girls (15 cases, 9.3%) was higher than that in boys (5 cases, 2.8%; *P* = 0.019), but the rate difference was not significant by age (0.450 < *P <*0.701). The egg-positive rate of *S. mansoni* was 16.0% in Khour Ajwal school, whereas none was found in the Elzaefa Elahamda or Sharrat schools (Table 4).

**Table 4:** **Univariate analysis of factors associated with** ***S. mansoni*** **infection among school children who participated in this study (n = 338)**

**Variable**	**Category**	**No. exam.**	**No. positive (%)**	**OR (95% ** **CI)**	***P-*** **value by logistic regression**	**Intensity of cases**	
						**Mean ± S.D. (log EPG)**	***P-*** **value**
Age in years	7-9	159	8 (5.0)	1		1.537 ± 0.960	0.736
	10-12	114	7 (6.1)	1.23 (0.43-3.49)	0.701	1.884 ± 0.922	
	13-15	65	5 (7.7)	1.56 (0.49-4.97)	0.450	2.053 ± 0.515	
Sex	Boys	176	5 (2.8)	1		1.117 ± 1.068	0.058
	Girls	162	15 (9.3)	3.47 (1.23-9.78)	*0.019*	2.011 ± 0.653	
School	Khour Ajwal	125	20 (16.0)	-	-	1.787 ± 0.844	-
	Elzaefa Elahamda	84	0 (0.0)				
	Sharrat	129	0 (0.0)				

OR, Odds ratio.

CI, confidence interval.

The intensity of *S. mansoni*-infected children was 1.787 ± 0.844 log EPG (range, 1–600 EPG; mean ± SD = 156 ± 176 EPG; Table 4). There was no significant difference by gender or age groups. Among infected children, 85.0% were classified as light or moderate infection (EPG <400), and 15.0% were considered heavily infected (EPG ≥400).

### Questionnaire responses for related risk factors and symptoms

Of the 200 students interviewed, 82 were *S. haematobium* egg-negative (41.0%) and 118 students (59.0%) were positive (Table 5). Regarding the frequency of water-contact, most of the children contacted water outside the house daily (74.5%), while others did so weekly (13.0%) or less often than weekly (12.5%). The egg-positive rates of students who contacted water increased with increased frequency of water contact (OR = 3.42, 95% CI = 1.41-8.27, *P* = 0.006), but no significant difference was seen between the frequency of water contact and infection intensity.

**Table 5:** **Distribution of** ***S. haematobium*** **egg positive rates according to water-contact patterns among 200 answered-school children in White Nile State, Sudan**

**Variables**		**No. (%)**	**No. of negative (%) (n = 82)**	**No. of positive (%) (n = 118)**	**OR**	**95** **%** **CI**	***P*** **-value by logistic regression**	**Intensity of positive cases**	
								**Mean ± S.D. (log EP10)**	***P-*** **value**
Contact for any reason									0.609
	Daily	149 (74.5)	51 (34.2)	98 (65.8)	3.42	1.41-8.27	*0.006*	1.099 ± 0.781	
	Weekly	26 (13.0)	15 (57.7)	11 (42.3)	1.30	0.42-4.03	0.645	1.320 ± 0.769	
	> Weekly	25 (12.5)	16 (64.0)	9 (36.0)	1			0.995 ± 0.871	
Collecting water									0.982
	Daily	147 (73.5)	53 (36.1)	94 (63.9)	1.89	0.87-4.13	0.109	1.118 ± 0.791	
	Weekly	22 (11.0)	13 (59.1)	9 (40.9)	0.74	0.25-2.23	0.590	1.076 ± 0.833	
	> Weekly	31 (15.5)	16 (51.6)	15 (48.4)	1			1.090 ± 0.757	
Taking baths									0.701
	Daily	137 (68.5)	46 (33.6)	91 (66.4)	4.26	2.02-9.00	*0.000*	1.132 ± 0.783	
	Weekly	22 (11.0)	8 (36.4)	14 (63.6)	3.77	1.27-11.21	*0.017*	0.945 ± 0.696	
	> Weekly	41 (20.5)	28 (68.3)	13 (31.7)	1			1.143 ± 0.907	
Swimming									0.658
	Daily	132 (66.0)	43 (32.6)	89 (67.4)	4.01	1.98-8.11	*0.000*	1.149 ± 0.784	
	Weekly	21 (10.5)	8 (38.1)	13 (61.9)	3.15	1.08-9.16	*0.035*	0.981 ± 0.710	
	> Weekly	47 (23.5)	31 (66.0)	16 (34.0)	1			1.008 ± 0.863	
Washing clothes									0.643
	Daily	124 (62.0)	41 (33.1)	83 (66.9)	1.94	0.97-3.88	0.062	1.154 ± 0.774	
	Weekly	31 (15.5)	19 (61.3)	12 (38.7)	0.60	0.24-1.53	0.288	0.966 ± 0.939	
	> Weekly	45 (22.5)	22 (48.9)	23 (51.1)	1			1.032 ± 0.753	
Wading the stream									0.307
	Daily	82 (41.0)	22 (26.8)	60 (73.2)	2.86	1.50-5.45	*0.001*	1.194 ± 0.825	
	Weekly	32 (16.0)	16 (50.0)	16 (50.0)	1.05	0.46-2.36	0.911	1.194 ± 0.756	
	> Weekly	86 (43.0)	44 (51.2)	42 (48.8)	1			0.962 ± 0.727	
Farming									0.533
	Daily	35 (17.5)	11 (31.4)	24 (68.6)	1.71	0.76-3.81	0.193	0.957 ± 0.786	
	Weekly	51 (25.5)	21 (41.2)	30 (58.8)	1.12	0.57-2.18	0.748	1.189 ± 0.786	
	> Weekly	114 (57.0)	50 (43.9)	64 (56.1)	1			1.133 ± 0.786	
Washing the vegetables									0.668
	Daily	23 (11.5)	8 (34.8)	15 (65.2)	1.41	0.56-3.52	0.467	1.031 ± 0.852	
	Weekly	30 (15.0)	11 (36.7)	19 (63.3)	1.30	0.58-2.92	0.532	1.253 ± 0.772	
	> Weekly	147 (73.5)	63 (42.9)	84 (57.1)	1			1.094 ± 0.780	
Fishing									0.157
	Daily	11 (5.5)	4 (36.4)	7 (63.6)	2.00	0.34-4.24	0.778	0.947 ± .0635	
	Weekly	7 (3.5)	4 (57.1)	3 (42.9)	0.39	0.11-2.36	0.392	1.943 ± 0.393	
	> Weekly	182 (91.0)	74 (40.7)	108 (59.3)	1			1.099 ± 0.790	

Next, we analyzed the risk factors for urinary schistosomiasis according to the water-contact patterns of the children. The frequencies of water-contact for taking baths (OR = 4.26, 95% CI = 2.02-9.00, *P* <0.001), swimming (OR = 4.01, 95% CI = 1.98-8.11, *P* <0.001) and wading the stream (OR =2.86, 95% CI = 1.50-5.45, *P* = 0.001) were related to the egg-positive rates, and positive rates were significantly higher among children who contacted water of daily versus those with weekly or less often than weekly contact (*P* ≤0.001). In contrast, there was no significant correlation between frequency of water contact and egg-positive rates in cases of collecting water, farming, fishing, or washing vegetables. There was no significant correlation between infection intensity and frequencies of water contact (0.157 < *P <*0.982; Table 5).

Table 6 summarizes symptoms related to egg-positivity and infection infectivity in urinary schistosomiasis. All of 200 students interviewed complained of at least one of the symptoms within the last 6 months. Hematuria (OR = 5.27, 95% CI = 2.59-10.73, *P* <0.001) and dysuria (OR = 3.56, 95% CI = 1.97-6.43, *P* <0.001) were significantly correlated with *S. haematobium* infection, whereas skin redness (OR = 0.49, 95% CI = 0.25-0.96, *P =* 0.038) was significantly related with non-infection. Skin itching, frequent fatigue, urticaria, diarrhea, fever, and weight loss were not significantly associated with urinary infection (0.058 < *P* <0.840). There was a significant correlation between infection intensity and hematuria (*P =*0.028).

**Table 6:** **Distribution of** ***S. haematobium*** **egg positive rates according to symptoms related with urinary schistosomiasis within 6 months among 200 answered-school children in White Nile State, Sudan**

**Symptoms**		**No. of answered (%)**	**No. of negative. (%) (n = 82)**	**No. of positive. (%) (n = 118)**	**OR**	**95% ** **CI**	***P*** **-value by logistic regression**	**Intensity positive cases**	
								**Mean ± S.D. (log EPG)**	***P*** **-value**
Skin itching									
	No	89 (44.5)	37 (41.6)	52 (58.4)	1			1.233 ± 0.867	
	Yes	111 (55.5)	45 (40.5)	66 (59.5)	1.04	0.59-1.84	0.883	1.016 ± 0.703	0.136
Skin redness									
	No	154 (77.0)	57 (37.0)	97 (63.0)	1			1.151 ± 0.791	
	Yes	46 (23.0)	25 (54.3)	21 (45.7)	0.49	0.25-0.96	*0.038*	0.928 ± 0.740	0.237
Frequent fatigue									
	No	107 (53.5)	47 (43.9)	60 (56.1)	1			1.169 ± 0.805	
	Yes	93 (47.5)	35 (37.6)	58 (62.4)	1.30	0.74-2.29	0.367	1.052 ± 0.763	0.420
Hematuria									
	No	132 (66.0)	70 (53.0)	62 (47.0)	1			0.962 ± 0.778	
	Yes	68 (34.0)	12 (17.6)	56 (82.4)	5.27	2.59-10.73	*0.000*	1.277 ± 0.762	*0.028*
Urticaria									
	No	199 (99.5)	82 (41.2)	117(58.8)	1			1.117 ± 0.785	
	Yes	1 (0.5)	0 (0.0)	1 (100.0)	0.00	0.00	1.000	0.477	0.419
Diarrhea									
	No	137 (68.5)	56 (40.9)	81 (59.1)	1			1.189 ± 0.775	
	Yes	63 (31.5)	26 (41.3)	37 (58.7)	0.98	0.54-1.80	0.958	0.942 ± 0.785	0.113
Fever									
	No	77 (38.5)	31 (40.3)	46 (59.7)	1			1.283 ± 0.785	
	Yes	123 (61.5)	51 (41.5)	72 (58.5)	0.95	0.53-1.70	0.866	1.002 ± 0.768	0.058
Dysuria									
	No	86 (43.0)	50 (58.1)	36 (41.9)	1			1.089 ± 0.796	
	Yes	114 (57.0)	32 (28.1)	82 (71.9)	3.56	1.97-6.43	*0.000*	1.121 ± 0.783	0.840
Weight loss									
	No	59 (29.5)	25 (42.4)	34 (57.6)	1			1.075 ± 0.733	
	Yes	141 (70.5)	57 (40.4)	84 (59.6)	1.08	0.59-2.01	0.798	1.126 ± 0.807	0.752

## Discussion

In Sudan, schistosomiasis is the most prevalent parasitic disease, and both urogenital and intestinal forms of schistosomiasis are common throughout the country with geographically varying degrees of prevalence [19]. According to previous reports on schistosomiasis, the overall prevalences of infections with *S. mansoni*, *S. haematobium*, or both among 6,122 children from 27 schools in the White Nile Province were 10.1%, 21.4%, and 4.5%, respectively [8]. The prevalence of *S. haematobium* in the Upper Nile region and South Darfur were 73% and 56.0%, respectively [9,10]. And the prevalence of *S. haematobium* in the River Nile State and Southern Kordofan State were 1.7% and 23.7%, respectively; however, there was no *S. mansoni* infection [11,12]. These data show that there was marked geographical variation in the prevalence of *Schistosoma* species infection. Depending on the survey sites, there was a big variability in *Schistosoma* species. The egg-positive rates of *S. haematobium*, *S. mansoni*, and both in the White Nile River basin were 45.0%, 5.9%, and 4.4%, respectively. The prevalence of *S. haematobium* infection in the area was lower than that in the Upper Nile region and South Darfur, Sudan [9,10]. However, compared with the results of the same area in 1996 [8], the *S. haematobium* egg-positive rate was increased more than two times. This study proved that the prevalence of *S. haematobium* infection was maintained at a high level in White Nile State. Schistosomiasis may have been neglected simply because it is more difficult to include chronic disability and illness into the agenda of Ministries of Health, Sudan, especially in the presence of more important diseases such as HIV/AIDS, TB and Malaria. Even if control successes were achieved in some areas of Sudan, they could not be sustained due to lack of funding [20].

Infection intensity reflects the number of worms infecting the individual, and is a more reliable marker of treatment success, which is defined as the removal of egg-laying worms. Also infection intensity is a better indicator of morbidity than prevalence in schistosomiasis [21]. According to the previous reports in Sudan, before praziquantel treatment, the intensities of *S. haematobium* were as high as 12.9 EP10 in White Nile Province [8], 25.5 EP10 at Gereida Camp in southern Darfur [13], and 87.7 EP10 (geometric mean) in Central Sudan [16]. Also, the intensities of *S. mansoni* were 97.7 EPG in White Nile Province [8] and 1.7 EPG (geometric mean) in the Geizira area of Central Sudan [22]. In the present study, more than 75% of the infected children were considered to have light or moderate infection of both *S. haematobium* (EP10 < 50) and *S. mansoni* infection (EPG <400), and the mean intensities of *S. haematobium* and *S. mansoni*-infected children were 57 EP10 (1.091 ± 0.744 log EP10) and 156 EPG (1.787 ± 0.844 log EPG), respectively. These data indicated that the intensities of *S. haematobium* and *S. mansoni* infection in the White Nile River basin had increased in comparison with previous results from 1996 [8], which were 12.9 EP10 and 97.7 EPG, respectively. Apparently, *S. haematobium* egg-positive rates in Khour Ajwal and Elzaefa Elahamda school children showed very high egg-positive rates (60.8-73.8%); thus, mass praziquantel treatment should be conducted in school-age children and high-risk groups of the population according to the WHO regulation guidelines urgently [18]. To implement the comprehensive schisotosomiasis control, besides mass therapy, the control plan should include various components such as health education, construction of a facility to supply drinking water and sanitary facilities, and vector control.

This study showed that there were significant differences in *S. haematobium* egg-positive rates between schools, but not between gender or age groups. In contrast, the egg-positive rates of *S. mansoni* were significantly different between boys and girls, but not among schools or age groups. Many reports showed that males usually have higher prevalence of schistosomiasis than females, and this was attributed to the observation that boys are more outgoing and adventurous in nature and they tend to play away from their homes more than their female counterparts [23]. These results may be due to various factors, such as proximity to the main stream, intermediate host snail distribution, environmental contamination with human excreta, human water-contact patterns and host-parasite relationships [24]. In the present study, the egg-positive rate of Sharrat village was much lower than that of Khour Ajwal and Elzaefa Elahamda villages. It may be due to several factors. Drinking water supply facilities such as artesian well and water filter systems were established in Sharrat village, but not in the other villages. Also the distance to the White Nile River of Sharrat village was 3 times longer (about 3 km) than that of Khour Ajwal and Elzaefa Elahamda villages (both within 1 km). Furthermore, Sharrat villagers may receive more health education about how to manage the pump operating clean water and the importance of clean water.

Schistosomiasis has been spread by contact with water that contains the larval parasites. In the present study, a higher frequency of water contact was significantly associated with higher positivity, but not with infection intensity. Similar results have been reported before: that the frequency of water-contact activities correlated with infective rates, but not with infection intensity [12,23,25]. The same patterns were observed in the present study. The frequency of water-contact activities of Sharrat school children was almost one third of Khour Ajwal and Elzaefa Elahamda schools, thus the egg-positive rate of Sharrat school children was significantly lower that of the other villagers. Next, we analyzed water-contact patterns to evaluate risk factors for the transmission of schistosomiasis. The frequency of water contact for bathing, swimming, and wading streams were significantly associated with the infection rate of urinary schistosomiasis, as reported by Rudge *et al.* [23]. However, others have described that fishing and watering vegetables were also closely associated with infection rates and the frequency of water-contact [25] and that the frequency of water-contact was not significantly associated with infection [26].

After infection by schistosomes, symptoms may develop, including fever, chills, cough, and muscle aches, within 1–2 months of infection. Later, without treatment, schistosomiasis can persist for years with abdominal pain, enlarged liver, blood in the stool and/or urine, and problems passing urine. Chronic infection can also lead to an increased risk of bladder cancer [1]. In this study, school children were the target subjects to be carefully looked at for evaluating the prevalence, risk factors and clinical manifestation of schistosomiasis, because schistosomiasis has detrimental effects on their growth and development, and the early diagnosis and treatment reduces the risk of severe disease and childhood disability [2,27]. Based on the questionnaire responses, hematuria and dysuria showed significant correlations with *S. haematobium* infection. Previous studies have shown that self-reported hematuria had the best correlation with urinary schistosomiasis in school-based control programs [23,28]; however, dysuria did not [29]. In the present study, the percentage of hematuria was significantly high at self-reporting cases (30%) in comparison to the authors’ observation (5%). This is why the conditions of hematuria were different from the authors’ own observation and self-reporting cases. We assessed hematuria immediately after collection of urine samples, whereas hematuria assessed by questionnaires was the accumulative result of recall of information over the last 6 months, which was not a real situation at that time point. Also, we observed that even uninfected children identified by microscopic examination reported schistosomiasis-related symptoms. The situation can be explained in several ways. First, self-reporting symptoms beside hematuria or dysuria were not specific for schistosomiasis, so they could suffer these symptoms arising from the other diseases during last 6 months. Second, the surveyed population were primary school children, who do not have the ability to define the various features of symptoms. Indeed, the results by questionnaire showed some bias and were different from the real features of symptoms. In case of skin redness, there were significantly unrelated to infection. This could be because there are many reasons for skin redness and it was not possible for primary school children to differentiate the etiology of the skin redness by schistosomiasis or other factors.

This study has some limitations. A single egg count used in the present study is less reliable in estimating the prevalence and infection intensities of schistosomiasis. The examination of two or more specimens per child would likely have resulted in higher estimates of total prevalence and intensity. Another limitation is the lack of information on adults, preschool children, and non-enrolled school-age children. Recent reports have suggested that adults and preschool children are at risk of schistosomiasis and can contribute to transmission [30]; thus, a population based survey is required to provide an overview of schistosomiasis in the target area.

Taken together, schistosomiasis, especially urinary schistosomiasis, is highly prevalent in the White Nile River basin of Sudan. Frequencies of water-contact by bathing, swimming, and wading the stream are risk factors for schistosomiasis in school children. Self-reported hematuria and dysuria are significantly associated with *S. haematobium* infection. These data provide essential information to facilitate targeted control measures to keep children healthy in White Nile State, Sudan.

## Conclusions

Our results showed that the prevalence of schistosomiasis is still high among school children along the White Nile River basin, Sudan, especially urinary schistosomiasis. Also, the frequencies of water contact for leisure and domestic activities are significantly associated with the prevalence of schistosomiasis. Thus, there is a need for an effective schistosomiasis control program to reduce contaminated water contact through the provision of sanitary and safe-water supply facilities as well as mass chemotherapy and health education.

## Abbreviations

EP10: Number of *S. haematobium* eggs per 10 mL of urine (EP10)
EPG: Number of *S. mansoni* egs per gram of stool (EPG)
